# Salivary testosterone measurement in women with and without polycystic ovary syndrome

**DOI:** 10.1038/s41598-017-03945-w

**Published:** 2017-06-15

**Authors:** Thozhukat Sathyapalan, Ahmed Al-Qaissi, Eric S. Kilpatrick, Soha R. Dargham, Joanne Adaway, Brian Keevil, Stephen L. Atkin

**Affiliations:** 1 Department of Academic Diabetes, Endocrinology and Metabolism, University of Hull, Hull, UK; 2 Department of Pathology, Sidra Medical and Research Center, Doha, Qatar; 3 Infectious Disease Epidemiology Group, Weill Cornell Medicine, PO Box, 24144 Doha, Qatar; 4 Department of Clinical Biochemistry, Wythenshawe Hospital, Manchester, UK; 5 Research Faculty, Weill Cornell Medicine, PO Box, 24144 Doha, Qatar

## Abstract

Clinical and/or biochemical hyperandrogenism is one of the diagnostic criteria for PCOS. An evaluation of the role of salivary testosterone (salT) and androstenedione (salA) for the diagnosis of PCOS was undertaken in a cross sectional study involving 65 women without PCOS and 110 women with PCOS fulfilling all 3 diagnostic Rotterdam criteria. Serum and salivary androgen measurements were determined by LC-MS/MS. salT and salA were significantly elevated in PCOS compared to controls (P < 001). No androgen marker was more predictive than another using ROC curves, but multiple logistic regression suggested salT was more predictive than free androgen index (FAI) (p < 0.01). The combination of salT or FAI identified 100% of PCOS women. PCOS women with both biochemical and clinical hyperandrogenism as opposed to clinical hyperandrogenism alone showed a metabolic phenotype (p < 0.05) and insulin resistance (p < 0.001). PCOS patients with an isolated elevated FAI showed increased insulin resistance compared to those with an isolated salT (P < 0.05). salT appeared to be at least as predictive as FAI for the diagnosis of the classical PCOS phenotype, and the combination of salT or FAI identified 100% of PCOS patients. This suggests that salT measurement by LC-MS/MS holds the promise of complementing existing laboratory tests as a means of assessing hyperandrogenemia.

## Introduction

Polycystic ovary syndrome (PCOS) is one of the most common endocrine disorders and affects 6–20% of reproductive-aged women^1–3^. Androgen excess is considered to be the central defect in PCOS women^4^, yet it is triggered by other factors, obesity and insulin resistance being frequently involved^5^. Hence measurements of androgens play a crucial role in the diagnosis of PCOS.

As liquid chromatography tandem mass spectrometry (LC-MS/MS) becomes more widely available in clinical practice, its advantages compared to immunoassays, including its specificity and sensitivity for the analyte being measured^6^, means it may be particularly suited to the measurement of typical low female androgen concentrations of testosterone and androstenedione^7^.

In respect of the diagnosis of PCOS, measurement of free or bioavailable testosterone is thought to be a superior means of assessing hyperandrogenemia in these patients as they both give a closer estimate of biologically active testosterone than total testosterone measurement alone^8^. However, measurement of these is relatively laborious, labor intensive and therefore expensive and so various formulae have been devised to estimate free testosterone, usually by combining more routinely measured serum testosterone and sex hormone binding globulin (SHBG) tests, such as the ‘free androgen index’^9^.

A high correlation has been shown between salivary testosterone (salT) and serum free testosterone measured by equilibrium dialysis in both men and women^10^, though salT is not directly comparable to serum free T due to T binding to saliva proteins, which substantially affects the low salT found in women. Saliva is potentially a sample type that will more directly assess a patient’s bioavailable testosterone which, combined with measurement by LC-MS/MS, would be especially applicable to PCOS patients, with possible utility in diagnosis, population research, population screening and treatment monitoring. In addition, such a test sample may prove to be at least as acceptable to patients as a venepuncture and could avoid a clinic attendance, especially if collection of multiple samples is required either as part of a clinical investigation or for research.

Given these potential advantages, this study was performed to establish if salT and salivary androstenedione (salA) measurements had a role in diagnosing PCOS, either instead of or in addition to existing laboratory tests.

## Materials and Methods

This was a cross sectional study involving 110 well characterised women with PCOS and 65 women without PCOS who presented sequentially to the Department of Endocrinology, Hull and East Yorkshire Hospitals NHS Trust and were recruited to the local PCOS biobank (ISRCTN70196169). All patients gave written informed consent. The Newcastle & North Tyneside Ethics committee approved this study. The diagnosis of PCOS was based on all three diagnostic criteria of the Rotterdam consensus, namely clinical and/or biochemical evidence of hyperandrogenism (Ferriman-Gallwey score >8; free androgen index >4 respectively), self reported oligomenorrhea (cycle length was greater than 35 days and 9 or fewer periods per year) or amenorrhea (no menses for 3 months or more) and polycystic ovaries on transvaginal ultrasound (≥12 antral follicles in at least one ovary or ovarian volume of ≥10 cm^3^)^5^. Polycystic ovaries were defined on transvaginal ultrasound if *(a)* one or both ovaries demonstrate 12 or more follicles measuring 2–9 mm in diameter, and/or *(b)* the ovarian volume exceeds 10 cm^3^ ^11^. Study participants had no concurrent illness, were not on any medication for the preceding 9 months and were not planning to conceive. Non-classical 21-hydroxylase deficiency, hyperprolactinaemia, Cushing’s disease and androgen-secreting tumours were excluded by appropriate tests. All women in the control women were healthy, had regular periods, no clinical or biochemical hyperandrogenemia, no significant background medical history and none of them were on any medications including oral contraceptive pills or over the counter medications. All women underwent a 75 g oral glucose tolerance test to exclude impaired glucose tolerance and type 2 diabetes. All PCOS and control women were Caucasian. Height, weight, waist circumference and body mass index (BMI) were performed according to WHO guidelines^12^. All study procedures and assays were performed in accordance with relevant guidelines and regulations.

### Collection and analysis of blood samples

Blood samples were taken within 5 minutes of saliva collection and were stored frozen at −80 °C pending analysis. Serum A and total serum T were measured by LC/MS/MS on an Acquity UPLC system coupled to a Quattro Premier XE mass spectrometer (Waters, Manchester, UK)^13^. Sex hormone binding globulin (SHBG) was measured by an immunometric assay with fluorescence detection on the DPC Immulite 2000 analyzer using the manufacturer’s recommended protocol. The free androgen index (FAI) was calculated as the total testosterone × 100/SHBG. Serum insulin was assayed using a competitive chemiluminescent immunoassay performed on the manufacturer’s DPC Immulite 2000 analyzer (Euro/DPC, Llanberis, UK). The analytical sensitivity of the insulin assay was 2 μU/ml, the coefficient of variation was 6%, and there was no stated cross-reactivity with proinsulin. Plasma glucose was measured using a Synchron LX 20 analyzer (Beckman-Coulter), using the manufacturer’s recommended protocol. The coefficient of variation for the assay was 1.2% at a mean glucose value of 5.3 mmol/liter during the study period. The insulin resistance was calculated using the HOMA method [HOMA-IR = (insulin × glucose)/22.5].

### Collection and handling of saliva samples

Participants were asked to spit or drool directly into a 4 mL sealable polystyrene tube and to provide at least 3 mL of saliva. Unstimulated saliva samples were used to avoid any assay interference. “Passive drool” technique was used for collection of saliva rather than ‘Salivette’^10, 14^. In order to avoid blood and other oral contaminations that may interfere with the assay, volunteers were requested to avoid dental work for 48 h prior to sample collection, avoid teeth brushing 2 h prior to sample collection, avoid eating for at least 1 h prior to sample collection and rinse their mouth with water not less than 10 min and not more than 15 min prior to sample collection. Saliva specimens were checked for blood contamination visually. The saliva samples were stored in −80 °C and were batch analysed for salT and salA using liquid chromatography-mass spectrometry (LC-MS/MS)^15^.

300 µL of saliva was mixed with 10 µL of a mixed internal standard (d7-(2,2,4,6,6,16,16)−4-androstene-3,17-dione and [^13^C_3_]-Testosterone in a 96-deep well plate. This was then transferred to a Biotage Isolute SLE+ 400 plate and the analytes eluted with dichloromethane. The eluant was evaporated to dryness then reconstituted in 40 (v/v) methanol. Further sample clean-up was carried out on a Waters Online SPE manager using XBridge C18 cartridges before a Kinetex C8, 3 × 100 mm 2.6 µm analytical column (Phenomenex, UK). LC-MS/MS analysis was performed using a Waters Acquity UPLC system coupled to a Waters Xevo TQS mass spectrometer.

We achieved a lower limit of quantification of 5 pmol/L for testosterone and 6.25 pmol/L for androstenedione. Recovery was 83–116% for both analytes and the assay was found to be linear up to 2500 pmol/L for testosterone and androstenedione. The inter-assay precision and bias were calculated at concentrations of 25, 50 and 100 pmol/L for each analyte. The CV was <4% for testosterone (bias <4%) and <7.5% for androstenedione (bias <6.5%) at all 3 concentrations. The intra-assay precision and bias were calculated at concentrations of 12.5, 25 and 50 pmol/L. The CV was <7.5% (bias <3.5%) for testosterone and <3% (bias <3.5%) for androstenedione at all three concentrations. Recovery experiments were conducted using certified reference material for testosterone and androstenedione (Cerillient, UK) spiked into saliva samples. Precision data was reported using PBS BSA spiked with testosterone and androstenedione in order that we could assess bias. Precision data using saliva pools for testosterone showed within batch CV of <4% at concentrations of 100 and 780 pmol/L. Androstenedione showed within batch CV of <8% at concentrations of 70 and 110 pmol/L respectively. Ion suppression was not detected at the retention times for either testosterone or androstenedione. Interference studies were carried out by injecting a wide range of steroids including epitestosterone, dihydrotestosterone and dehydroepiandrosterone at supraphysiological concentrations and no interference was detected.

### Statistical analysis

Data trends were visually evaluated for each androgen and non-parametric tests were applied on data that violated the assumptions of normality when tested using the Kolmogorov-Smirnov Test. Accordingly, comparative analysis evaluating androgen levels between PCOS cases and controls was performed using the non-parametric Mann-Whitney test. Pearson’s correlations were also estimated to assess any linear relationship between different androgens. Finally, a multivariable logistic regression adjusting for age and BMI was conducted to assess the effects of different androgens in diagnosing PCOS. Significance was defined at α = 0.05. All analyses were done using IBM-SPSS version 24.0. All values are given as (median ± IQR) unless specified.

## Results

The baseline demographics of patients are given in Table 1. PCOS women were significantly younger (25.5 ± 10.0 years) than the controls (32.0 ± 12.0 years). Their body mass index (BMI), waist and hip circumferences were also significantly higher (p < 0.001) than the controls (Table 1). Patients with PCOS showed greater insulin, HOMA-IR, 2 hour glucose post oral glucose tolerance test (OGTT) values (p < 0.001) (Table 1). All 110 PCOS women were categorized as classical phenotype according to the combination of all 3 major Rotterdam’s Consensus Criteria.

**Table 1:** Comparison of various anthropometric and hormonal parameters between women with PCOS and controls.

Parameters	Controls		PCOS		p value
	Median	IQR	Median	IQR	
Age (years)	32.00	12.00	25.50	10.00	0.008*
BMI (kg/m^2^)	25.00	6.20	33.00	10.50	<0.001*
Waist Circumference (cm)	78.00	15.00	101.00	23.30	<0.001*
Hip Circumference (cm)	101.00	15.50	116.00	19.30	<0.001*
Salivary Testosterone (pmol/L)	13.11	10.00	18.48	15.00	<0.001*
Salivary Androstenedione (pmol/L)	142.89	95.00	165.76	118.00	0.001*
Total Testosterone (nmol/L)	1.00	0.50	1.25	0.70	<0.001*
SHBG (nmol/L)	44.00	26.00	27.00	22.00	<0.001*
FAI	2.27	2.04	4.26	4.71	<0.001*
Androstenedione (nmol/L)	7.40	5.90	40.31	7.90	0.001*
Salivary Testosterone/Androstenedione ratio	0.08	0.05	0.10	0.07	<0.001*
Serum Testosterone/Androstenedione ratio	0.12	0.08	0.12	0.09	0.918
Baseline Glucose (mmol/L)	4.60	0.50	4.65	0.40	0.002
2 Hour Glucose (mmol/L)	5.00	1.30	5.65	1.70	<0.001*
Insulin (μIU/ml)	6.40	4.08	13.75	11.69	<0.001*
HOMA-IR	1.34	0.98	2.86	2.47	<0.001*

*BMI – Body Mass Index; FAI – Free Androgen Index; HOMA-IR – Homeostasis model of assessment – insulin resistance: IQR – inter quartile range. To convert values for glucose to milligrams per deciliter, divide by 0.056. To convert values for insulin to picomoles per liter, multiply by 6. To convert values for testosterone to nanograms per deciliter, divide by 0.03467. To convert values for SHBG to micrograms per deciliter, divide by 34.7*. Demographics of the 175 subjects involved in the study, 65 women without PCOS and 110 women with PCOS. The diagnosis of PCOS was based on all three diagnostic criteria of the Rotterdam consensus, namely clinical and/or biochemical evidence of hyperandrogenemia (Ferriman-Gallwey score > 8; free androgen index > 4 respectively), oligomenorrhea or amenorrhea and polycystic ovaries on transvaginal ultrasound. These women therefore represented the phenotype with the greatest metabolic features.

All androgens including salT (PCOS vs. control, 18.48 ± 15.00 vs. 13.1 ± 10.00 pmol/L), salA (165.76 ± 118.00 vs. 142.89 ± 95.0 nmol/L), serum T (1.25 ± 0.70 vs. 1.0 ± 0.5 nmol/L), serum A (40.31 ± 7.9 vs. 7.40 ± 5.90 nmol/L), FAI (4.26 ± 4.71 vs. 2.27 ± 2.04), and the salT/salA ratio (0.10 ± 0.07 vs. 0.08 ± 0.05) were all significantly elevated in PCOS compared to control (p < 0.001). Among PCOS women, salT did not significantly correlate linearly with either FAI (Pearson’s R = 0.071, p = 0.512) or with serum T (Pearson’s R = 0.004, p = 0.970). salA showed a modest correlation with serum A (Pearson’s R = 0.319, p < 0.01) (data not shown).

When those PCOS women with both clinical and biochemical hyperandrogenism (raised FAI or serum T) were compared to those patients with clinical hyperandrogenism alone (normal FAI and serum T), women with PCOS and biochemical hyperandrogenism had features of a more metabolic phenotype with a greater BMI, waist and hip circumference (p < 0.05), and both higher insulin and insulin resistance levels (p < 0.001) (Table 2).

**Table 2:** Comparison of PCOS women with both biochemical and clinical hyperandrogenism versus PCOS women isolated clinical hyperandrogenism.

Parameters	Clinical and biochemical hyperandrogenism n = 58	Clinical hyperandrogenism n = 52	P-value
Salivary Testosterone (pmol/L)	81.69 (357.11)	16.76 (8.21)	<0.001
BMI (kg/m^2^)	35.94 (7.44)	31.76 (7.55)	<0.001
Waist Circumference (cm)	103.76 (17.23)	96.57 (15.46)	0.035
Hip Circumference (cm)	121.57 (16.25)	113.05 (16.96)	0.036
Baseline Glucose (mmol/L)	4.96 (1.33)	4.74 (0.54)	0.329
2 Hour Glucose (mmol/L)	6.46 (2.60)	5.53 (1.28)	0.187
Insulin (μIU/ml)	20.92 (17.72)	11.65 (6.86)	<0.001
HOMA-IR	5.30 (7.97)	2.53 (1.73)	<0.001

*Values in (mean (SD)). HOMA-IR – homeostatic model assessment – insulin resistance. BMI – Body Mass Index*. Comparison of PCOS women with both biochemical and clinical hyperandrogenism (raised serum T and/or a raised FAI) compared to those PCOS women with clinical hyperandrogenism (no elevated serum androgen level). As shown in this analysis, PCOS women with both biochemical and clinical hyperandrogenism showed a more metabolic phenotype compared to those PCOS women with clinical hyperandrogenism alone.

The combination of either a raised salT or a raised FAI identified 100% of PCOS patients. Within this group, there were 16 FAI positive and salT negative PCOS patients, and 12 FAI negative and salT positive PCOS patients. As shown in Table 3, although there was no difference in age, BMI, waist or hip circumference, the patients with a raised FAI alone showed other features usually associated with a metabolic phenotype by having elevated 2 hour glucose, insulin and HOMA-IR values (p < 0.05).

**Table 3:** Comparison of PCOS women with an elevated isolated free androgen index versus an elevated isolated salivary testosterone.

Parameters	High FAI, normal salT n = 16	High salT, normal FAI n = 12	P-value
BMI (kg/m^2^)	35.25 (5.92)	32.13 (7.69)	0.051
Waist Circumference (cm)	101.02 (12.20)	98.23 (17.52)	0.255
Hip Circumference (cm)	120.54 (12.85)	116.69 (15.21)	0.115
Baseline Glucose (mmol/L)	4.65 (0.38)	4.77 (0.42)	0.921
2 Hour Glucose (mmol/L)	6.04 (1.62)	5.07 (1.39)	0.034
Insulin (μIU/ml)	16.42 (11.31)	10.68 (5.87)	0.020
HOMA-IR	3.46 (2.45)	2.28 (1.28)	0.037

*Values in (mean (SD)). salT – salivary testosterone. FAI – free androgen index. HOMA-IR – homeostatic model assessment – insulin resistance. BMI – Body Mass Index*. Comparison of PCOS women who had an elevated FAI alone (salT not elevated) and those with an elevated salT (FAI not elevated). As shown in this analysis, women with a raised FAI alone compared to the raised sal T alone had a more metabolic phenotype.

**Figure 1:**
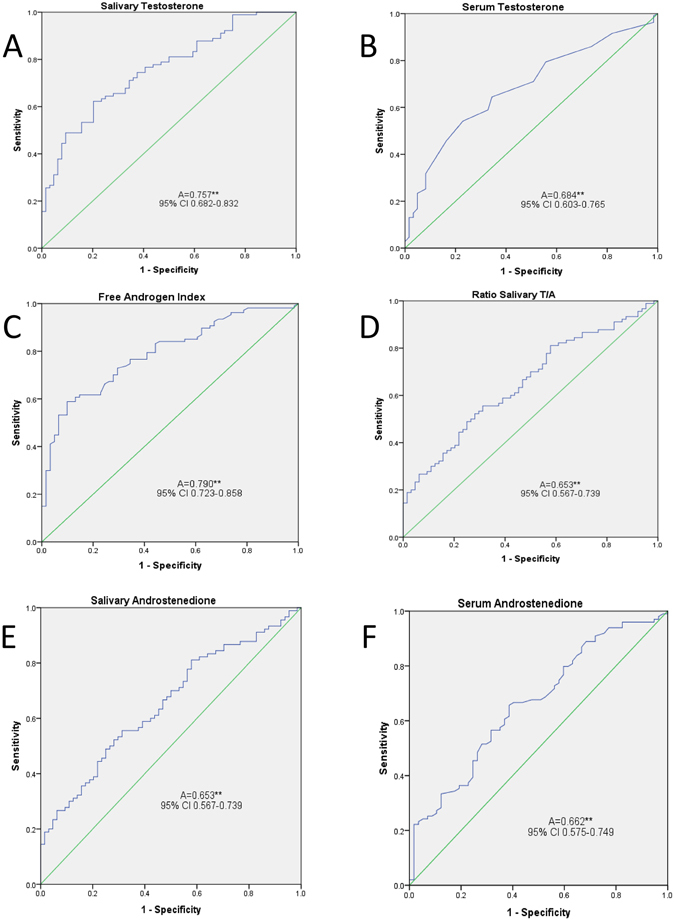
Receiver operating curves for salivary and serum androgens. Receiver operator curves for (**A**). Salivary testosterone, (**B**). Serum testosterone, (**C**). Free androgen index (FAI), (**D**). Salivary testosterone/salivary androstenedione ratio, (**E**). Salivary androstenedione, (**F**) Serum androstenedione. These show that FAI, salivary testosterone and (less so) serum testosterone were “good” predictors of a diagnosis of PCOS, whilst the salivary testosterone/salivary androstenedione ratio, salivary androstenedione and serum androstenedione were “fair” predictors of a diagnosis of PCOS.

**Figure 2:**
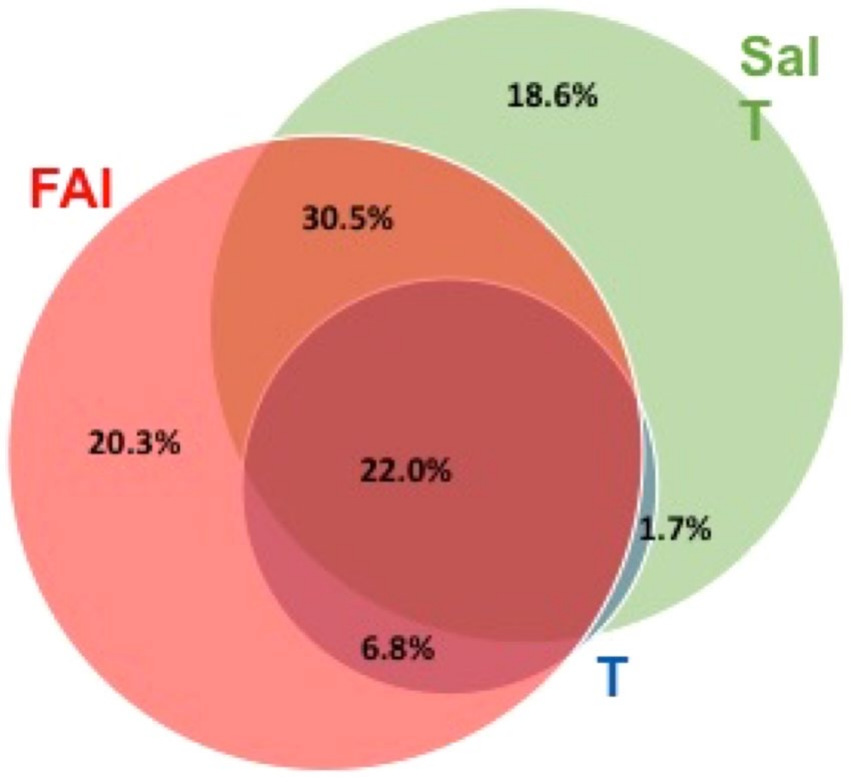
Venn diagram of elevated Salivary Testosterone, Serum Testosterone, and Free Androgen Index in the diagnosis of PCOS. Venn diagram of the percent of PCOS patients with a raised salivary testosterone (salT), a raised free androgen index (FAI) or a raised serum testosterone (T) showing that 100% of patients could be accounted for by a combination of the salivary testosterone and FAI, but indicating that not one single androgen measure would encompass the diagnosis of PCOS in all patients

The diagnostic upper thresholds for salT, serum T, and FAI were calculated using the 95^th^ percentile of the controls for the receiver operator curves (ROC), Fig. 1. These threshold values for serum T and FAI coincided exactly with the local laboratory reference range at 1.9 nmol/l and 4.0 respectively. Using these, the proportion of PCOS patients with elevated salT (>19.9 pmol/L), FAI and serum T were 48.9%, 53.3%, and 18.7%, respectively (Figure 2). The areas under the ROC curves showed no significant differences between androgen markers in predicting PCOS, although the trend was for FAI (0.79) and salT (0.76) to be better than serum T, salA, serum A and the salT:salA ratio (0.68, 0.65, 0.66, and 0.65 respectively).

As individual tests, an age and BMI adjusted multiple logistic regression suggested salT (OR = 1.074; 95% CI 1.017–1.133; p = 0.010) to be the better predictive measure than FAI (OR = 1.236; 95% CI 0.982–1.556; p = 0.072) (Table 4). salT (p = 0.010) and FAI (p = 0.041) were both significantly individually superior to serum T (p > 0.200) (Tables 5 and 6, respectively) in predicting PCOS.

**Table 4:** Multiple logistic regression analysis for the androgen prediction of PCOS including age, body mass index, salivary testosterone and free androgen index.

Parameters (units)	Adjusted		
	Odds Ratio	95% CI	P-value
Age (years)	0.961	0896–1.032	0.27
Body mass index (kg/m^2^)	1.169	1.078–1.266	<0.001
salT (nmol/L)	1.074	1.017–1.133	0.01
FAI	1.236	0.982–1.556	0.072

Logistic regression taking into account age and body mass index (BMI) showing that in this model salivary testosterone (salT) is significantly more predictive of PCOS than the free androgen index (FAI) or serum T (Tables 5 and 6, respectively), and that FAI was more predictive than serum T (C).

**Table 5:** Multiple logistic regression analysis for the androgen prediction of PCOS including age, body mass index, salivary testosterone and serum testosterone.

Parameters (units)	Adjusted		
	Odds Ratio	95% CI	P-value
Age (years)	0.951	0.888–1.017	0.144
Body mass index (kg/m^2^)	1.201	1.111–1.299	<0.001
salT (nmol/L)	1.075	1.018–1.135	0.010
Serum Testosterone (nmol/L)	1.568	0.701–3.507	0.274

Logistic regression taking into account age and body mass index (BMI) showing that in this model salivary testosterone (salT) is significantly more predictive of PCOS than the free androgen index (FAI) or serum T (Tables 5 and 6, respectively), and that FAI was more predictive than serum T (C).

**Table 6:** Multiple logistic regression analysis for the androgen prediction of PCOS including age, body mass index, free androgen index and serum testosterone.

Parameters (units)	Adjusted		
	Odds Ratio	95% CI	P-value
Age (years)	0.973	0.913–1.038	0.410
Body mass index (kg/m^2^)	1.182	1.092–1.279	<0.001
FAI	1.303	1.011–1.678	0.041
Serum Testosterone (nmol/L)	1.305	0.522–3.261	0.569

*salT – salivary testosterone. FAI – Free Androgen Index*. Logistic regression taking into account age and body mass index (BMI) showing that in this model salivary testosterone (salT) is significantly more predictive of PCOS than the free androgen index (FAI) or serum T (Tables 5 and 6, respectively), and that FAI was more predictive than serum T (C).

The combination of serum T, FAI and serum androstenedione (upper reference limit 7.4 nmol/L) identified 75% of PCOS subjects. None of the women in the control group had either a raised salivary testosterone or salivary androstenedione.

FAI was significantly associated with 2 hour glucose, SHBG and insulin (0.379, −0.498 and 0.415 respectively, p < 0.001). There was no significant correlation with insulin or HOMA-IR for any of the salivary androgens.

## Discussion

In this study salT appeared to be at least as good as the FAI in identifying patients with PCOS, and both were better than total serum T alone. However, while no single measure was predictive for all women with PCOS, all were identified by a combination of having either a raised FAI or a raised salT concentration.

The areas under the ROC curves showed no significant differences between androgen markers in predicting PCOS, although the trend was for FAI (0.79) and salT (0.76) to both better the serum T, salA, serum A and the salT:salA ratio. However, the multiple logistic regression model (taking into account patient age and BMI) showed salT to be statistically significant in being the more predictive marker. Economically, there is no additional cost to measure salT simultaneously with serum T when using LC-MS/MS.

The definition of an “elevated” salT was derived from the 95^th^ percentile of the ROC curve and clearly a larger population study would be needed to confirm this for routine laboratory practice; however, it was salient that the elevated FAI and serum T derived from the 95^th^ percentiles of the ROC curves in this study matched exactly those of the local reference laboratory, giving reassurance of the salT cut off point.

For those PCOS women with both biochemical hyperandrogenism (including those with a raised salT) and clinical hyperandrogenism versus clinical hyperandrogenism alone, women with PCOS and both biochemical and clinical hyperandrogenism had a more metabolic phenotype with higher insulin and insulin resistance levels that may have reflected the greater BMI, waist and hip circumference rather than being a specific and distinct PCOS subtype. Clinical hyperandrogenism has been shown to correlate poorly with biochemical hyperandrogenism^16^, though is helpful in the diagnosis of PCOS, and may differ ethnically^17^. Clinical and biochemical phenotype are also genetically determined. Indeed, polymorphisms of the gene putatively involved in the pathogenesis of polycystic ovary syndrome are associated with worse metabolic and clinical characteristics. For instance, Gly972Arg IRS-1 polymorphism is associated with higher Ferriman-Gallwey score, serum androgenemia and insulinemia. These effects are partially counterbalanced by another polymorphism, Lys121Gln, when it is present^18, 19^.

There was heterogeneity with some PCOS women having a positive salT, but normal FAI, and *vice versa*. Comparison between these 2 groups of a raised isolated salT versus those with a raised isolated FAI showed that those with the raised FAI had more features of a metabolic phenotype with a significantly elevated two hour glucose, insulin levels and insulin resistance suggesting that these may be identifying different PCOS subtypes. Those with a raised FAIs may reflect the role of SHBG that is reduced by insulin resistance that will increase FAI, and that may in turn increase insulin resistance. If salT and FAI were measuring different populations then this would then be in accord with the fact that a combination of both salT or FAI together identified 100% of the PCOS patients in this study. Therefore, in cases of PCOS of diagnostic uncertainty salT may add value for confirmation of the diagnosis, and as a test may be warranted to be more widely available in clinical practice. By comparison, no patient had an elevated serum T without also having either a raised FAI and/or a raised salT. Age and BMI adjusted analyses showed that the salT and FAI were both significantly superior in predicting the diagnosis of PCOS to serum T and likely should be the preferred androgen tests to be undertaken.

As noted, salT is not directly comparable to serum free T^10^, it is possible FAI and salT could be identifying two different subgroups of PCOS. There was an association between BMI and FAI in women with PCOS, possibly driven by a reduced sex hormone binding globulin (SHBG) concentration^20^. SHBG is considered to be a surrogate marker for insulin resistance^21^. Women with central obesity usually have lower serum SHBG concentrations in comparison with their age- and weight-matched counterparts with peripheral obesity^22^. This is dependent on higher circulating insulin which exerts an inhibitory effect on SHBG synthesis by the liver^23^, though SHBG may also be influenced by genetic variability^24^. Since salT is not bound to SHBG, variability of SHBG would not affect salT levels^25^.

The salT/salA ratio was significantly elevated in PCOS patients whilst the serum T/serum A ratio was not. However, the ROC analysis showed that salA, serum A and the salT/salA ratio trended towards being equally poorly predictive for PCOS by comparison to either salT or FAI alone. This is surprising given the strong predictive value of serum A for PCOS that was reported recently^26^, though the population in that study encompassed other PCOS phenotypes rather than the single classification here. Both salivary T and A were elevated in the saliva of PCOS patients with the relative greater increase in T being responsible for the increased salivary T/A ratio for PCOS compared to controls. The unequal elevation of T compared to A in saliva is thought to be due to salivary binding proteins for T, particularly in women^10^. In this current study, serum T, FAI and serum A defined hyperandrogenemia was found in 75% of PCOS subjects this being lower than the 90% reported by others^22^. FAI was significantly associated with an adverse metabolic phenotype and correlated to 2 hour glucose, SHBG and insulin, and A^26, 27^.

We found the testosterone and androstenedione concentrations in saliva to be lower and to have no correlation with the total testosterone and androstenedione in serum. Saliva contains only the free unbound fraction of testosterone which freely diffuses across capillaries and salivary ducts and is unaffected by saliva flow rates^28^. salT is therefore unaffected by variations in circulating SHBG and albumin unlike serum total testosterone. Measuring salT concentrations therefore provides an opportunity to directly assess ‘tissue’ free testosterone concentrations, which in turn may be a more accurate indicator of overall androgenicity and an alternative to serum free (free-T) or bio available-T. The latter are usually derived from mathematical formulae estimates based on association constants of testosterone with binding proteins and their relationship to measured free-T, and their clinical usefulness have been questioned^29^.

Salivary testosterone to androstenedione ratio has shown to be associated with adverse metabolic phenotypes in women with PCOS^30^. However, in our study we did not find any significant correlation of salT/salA ratio with any of the parameters. This could be due to the fact that the population in our study was homogenous comprising only the “classical phenotype” fulfilling all the three Rotterdam Consensus Criteria. As we have detailed, the clinical and biochemical phenotypes are genetically determined and this may also influence the entry of the steroids from blood into saliva that may be an active process^10^. Whilst the correlation of salivary androgens to metabolic phenotype was undertaken, these parameters were not related to the diagnosis of PCOS as described here.

This study maybe considered limited in scope as only the “classical” PCOS phenotype associated with all its metabolic features in a homogeneous Caucasian population within a narrow age range were included. A larger study will be needed to explore the heterogeneity of PCOS, where women may be positive for one androgen measure but not others, as well as investigating whether our findings hold true in different ethnic populations. Whilst all women fulfilled the ultrasound diagnostic criteria for PCOS with more than 12 peripherally arranged follicles and/or ovarian volume of 10 cm^3^, precise measurement of ovarian volume and absolute follicle count and maturity could not be undertaken for correlation with testosterone levels, indeed, ovarian MRI is suggested to be the better imaging modality for this purpose^31^. The highly significant differences seen between the control and PCOS patients should nonetheless make it unlikely that this biased the overall results reported. The PCOS and control group were also not age and BMI matched although this should be at least partially addressed by the multiple logistic model, which included both these parameters in the analysis.

In conclusion, salT appeared to be at least as predictive as FAI for the diagnosis of the classical PCOS phenotype in patients who fulfil all 3 of the Rotterdam Consensus Criteria, and whilst there was heterogeneity in the elevation of androgens, the combination of a raised salT or FAI identified 100% of PCOS patients participating in this study. This means that salT measurement by LC-MS/MS holds the promise of complementing existing routine laboratory tests to assess hyperandrogenemia in PCOS particularly in cases where there is diagnostic uncertainty.
